# Comparative Proteomic Analysis by Isobaric Tags for the Relative and Absolute Quantification Reveals the Responses of Tobacco (*Nicotiana tabacum* L.) Roots to Different Soil Types

**DOI:** 10.3389/fpls.2022.847388

**Published:** 2022-04-25

**Authors:** Jialiang Li, Rui Yang, Yonglei Jiang, Shubin Sun, Junying Li, Hao Gu, Ying Lin, Xianxue Luo, Chenggang He, Yi Chen

**Affiliations:** ^1^Yunnan Academy of Tobacco Agricultural Sciences, Yuxi, China; ^2^College of Tobacco Science, Yunnan Agricultural University, Kunming, China; ^3^Shiyan Branch of Hubei Tobacco Company, Shiyan, China; ^4^Research Center of Hubei Tobacco Industrial Co., Ltd., Xiangyang, China; ^5^Hunan Zhangjiajie Municipal Tobacco Co., Zhangjiajie, China

**Keywords:** iTRAQ, tobacco roots, soil type, proteomics, KEGG

## Abstract

Tobacco (*Nicotiana tabacum*) root affects the yield and quality of tobacco leaves. To gain insight into the responses of the tobacco root system to different soil types, we integrated morphological characteristics, the physiological index, the metabolic pathways of the root system, and the aboveground biomass of tobacco cultivated in limestone soil (LS), paddy soil (PS), and red soil (RS). Compared with plants growing in LS and PS, the chemical composition of tobacco leaves in RS tended to be coordinated. Red soil facilitated the accumulation of aboveground and belowground biomass of flue-cured tobacco and had the most significant effect on the dry matter quality of the roots. In addition, it promoted an increased root length, root surface area (RSA), root volume, and a higher number of root forks and improved root vigor and nitrate reductase (NR) activity; however, the activities of superoxide dismutase (SOD) and peroxidase (POD) were decreased. We studied differentially the abundant proteins (DAPs) of the flue-cured tobacco roots cultivated in different soil types by isobaric tags for the relative and absolute quantification (iTRAQ) of the proteomic profiles of cultivar. In total, 699, 650, and 569 differentially abundant proteins (DAPs) were identified from limestone soil (LS) vs. PS, LS vs. RS, and PS vs. RS, respectively, including 412/287, 291/359, and 323/246 up-/downregulated proteins, respectively. These DAPs were mainly involved in starch and sucrose metabolism, phenylalanine metabolism, the biosynthesis of secondary metabolites, microbial metabolism in different environments, and ribosomes. The parallel reaction monitoring (PRM) and quantitative reverse transcription PCR (qRT-PCR) analysis showed that the results of the iTRAQ proteomics were reliable. Overall, our study facilitates a new understanding of the responses of tobacco roots to different soil types at the protein level.

## Introduction

The root system in an important vegetative organ of plants, providing support, water and nutrient absorption storage, and the secretion of metabolites (Zhang and Lu, 2021), making it an integral part of plants (He and Wang, 1980). As the root system can be used as an index of plant growth and development (Nagel et al., 2009; Hou et al., 2018), investigating the mechanisms underlying root growth and distribution, root changes in different growth stages, and different environments can facilitate crop production. According to a previous study, plant roots can synthesize 18 kinds of amino acids, including not only 15 protein amino acids but also γ-aminobutyric acid and alvanic acid (Wu et al., 2021).

The root system of flue-cured tobacco is the main place where tobacco plants secrete secondary metabolites, such as nicotine and plant hormones (Wang et al., 2016). The amount of nicotine synthesized by flue-cured tobacco roots accounts for 99% of the total nicotine produced (Xu et al., 2004). As the root system of flue-cured tobacco directly affects the coordination of chemical components (Xie et al., 2010), it has indirect effects on the yield and quality of flue-cured tobacco. Hou (2003) found that the root weight of flue-cured tobacco was positively correlated with plant height and maximum leaf length and width, and compared with tobacco plants with a lower average root weight, the incidence and disease index of flue-cured tobacco with higher root weight were decreased. In addition, the yield, average price, proportion, and output value of middle- and high-grade tobacco, all increased with the increasing root weight. When the root weight per plant was 133.2 g, the chemical composition in leaves tended to be coordinated, with the highest levels in reducing sugars, total sugars, and Schmuck’s value. In addition, the protein and total nitrogen levels decreased, facilitating an increased tobacco quality. Hu (2015) showed that the root morphology of flue-cured tobacco affects soil water and nutrient absorption and use efficiency, which is directly related to the fixation effect of roots. Huang (2008) reported that the main part of nutrient uptake by roots is the young root tip, which is not yet embolized nor lignified.

Flue-cured tobacco can produce numerous adventitious roots after transplanting, which are younger than the main and lateral roots and are the main sites of absorption and synthesis. When tobacco plants are subjected to nutritional stress, adventitious roots will compensate or over-compensate nutrient losses (Xu and Fan, 1997; Zeng et al., 2015; Sascha et al., 2020).

The effect of soil type on the root growth of flue-cured tobacco is greater than that of soil moisture and temperature. Ma et al. (2003) studied the characteristics of tobacco root development under different soil types and showed that the root dry weight of flue-cured tobacco increased with throughout the growth period and peaked at the dome stage. Based on their results, the root development characteristics of flue-cured tobacco varied across 10 different soil types, along with the effects of the different soil types on the growth, development, and distribution of flue-cured tobacco.

In another study, different nitrogen forms had different effects on microorganisms in the yellow cinnamon soil and fluvo-aquic soil, thereby differing in their impacts on the growth and development of roots and the absorption and use of nitrogen (Liu et al., 2004). Yellow cinnamon soil and fluvo-aquic soil are dominated by clay and sand-grained complexes, respectively (Shen, 1994). In contrast to fluvo-aquic soil, the nutrients in yellow cinnamon soil are not easy to be mineralized. Gao et al. (2007) proposed that overly sandy or sticky soils are not conducive to the growth of flue-cured tobacco root systems. Chen R. X. et al. (2012) studied the effect of soil type on the growth of flue-cured tobacco; the authors used paddy soil (PS), purple soil, and red loam and found that the purple soil had the highest content of total potassium, whereas PS was rich in organic matter, and the pH in red soil (RS) was most suitable for the growth of flue-cured tobacco. In addition, the soil texture and water-holding capacity differed among the different soil types, resulting in differences in nutrient uptake.

Appropriate field water conditions can promote root growth, whereas drought or waterlogging will seriously affect the root growth. Yang et al. (2017), in a pot experiment, found that the root system of flue-cured tobacco developed slowly at a field water content of 20%, whereas at 80%, root vigor was impeded; the most suitable field water content was 50%. Han and Zhang (1992) showed that field water content of 60%, 80% and 60% respectively is beneficial to the root growth of flue-cured tobacco, in the three growth stages of flue-cured tobacco: root elongation, prosperity and maturity.

Tobacco soil acidity and alkalinity can also impact the growth and development of flue-cured tobacco roots, consequently affecting mineral absorption and nicotine synthesis (Chen et al., 2010). Xu et al. (2004), in a hydroponic experiment, found that the dry weight and volume of the root system increased with increasing pH values, albeit only within a range of 4.5–7.5; at higher levels, the dry weight and volume of the root system decreased. At a pH of 8.5, the active absorption area, total absorption area, root vigor were lowest.

Zhou et al. (1999) studied the adaptability of different flue-cured tobacco varieties to various rhizosphere pH levels and observed differences among the varieties; additionally, the root system could self-regulate the rhizosphere pH. When the soil environment is too acidic, with a pH as low as 4.5, root growth is seriously inhibited. Guo et al. (2000) used the sand culture method to explore the effect of pH on flue-cured tobacco roots; within a certain range, with increasing pH, the ATP content and respiration of roots also increased.

The term proteome was originally put forward by the Australian scholars Wilkins et al. (1996) at a conference in 1994 and refers to “a complete set of proteins expressed by a genome” and which has a certain diversity and variability. The proteome as such constantly changes over time and depending on the conditions (Swinbanks, 1995; Meng and Huo, 2005). Proteomics involves the study of proteomes to clarify the existence modes, functions, and activity modes of all proteins in the cells, tissues, and bodies (Chen et al., 2005). Since the proteome is constantly changing, it is difficult to analyze all proteins in the organism; therefore, currently, proteomics mainly studies the changes in cell protein composition in different periods. Studies have also investigated proteins in terms of protein post-translational modification, expression, structure, functional model, and protein–protein interaction (Anderson et al., 2000; Sun et al., 2005; Ruan et al., 2006; Cai, 2014).

Isotope-labeled relative and absolute quantitative isobaric tags for relative and absolute quantitation (iTRAQ)/tandem mass tag (TMT) is a new quantitative technique for the high-throughput screening of proteins. The tags used in the analysis can be divided into reflection group, equilibrium group, and reporter group; they are used for labeling 8–10 samples at the same time, and almost all proteins in the sample can be labeled (Xiang et al., 2010; Ruiling et al., 2016; Chen et al., 2017). The underlying principle is to combine the “isotope tag” peptide tag with liquid chromatography with tandem mass spectrometry (LC-MS/MS), and through the signal intensity difference of “tag” in MS/MS (MS2), the relative quantitative analysis of each protein expression level in multiple groups of samples can be performed (Zhang et al., 2014). The iTRAQ/TMT technology has the characteristics of a high labeling efficiency, a simpler labeling process, and a wide tagging range (Yao, 2015).

Recently, tobacco has been intensively studied, and as a new method, the proteomics technology has achieved important results. For example, Lin et al. (2012) compared four total protein extraction methods, namely, conventional lytic solution, tricarboxylic acid (TCA)/acetone precipitation, trizol extraction, and the phenol method, using the growing roots of cultivated tobacco variety K326, and tested the effect of protein separation by subsequent 2-DE. The results showed that the trizol extraction and the phenol method were most suitable for follow-up analysis. Cai et al. (2015) used comparative proteomics to study the protein expression of flue-cured tobacco leaves in Kunming and Lijiang. The authors identified 33 differentially abundant proteins, among which those related to defense/stress resistance, protein synthesis, and mineral metabolism were highly expressed in Kunming tobacco leaves, whereas those related to enhanced photosynthetic efficiency were highly expressed in tobacco leaves in Lijiang. Chen Z. Y. et al. (2012) applied proteomics to study the effects of different ultraviolet radiation levels on physiological metabolism and regulation pathways of flue-cured tobacco planted on a low-elevation plateau, whereas Lei et al. (2016) used the iTRAQ technique to quantitatively study the protein expression and bioinformatics in tobacco leaves planted in two different regions. Based on the results, the proteome expression patterns of tobacco leaves in the same ecological region were different and were involved in the differential expression of proteins related to photosynthesis and secondary metabolite biosynthesis, resulting in the formation of different aroma substances in tobacco leaves from the different regions.

In this context, the purpose of this study is to understand the genetic regulation mechanism of tobacco roots to different soil types at the protein level. This work will provide new insights into the response of tobacco roots to soil types and into the key proteins, with the overall aim to improve the tobacco yield and quality.

## Materials and Methods

### Plant Material and Growth Conditions

**FIGURE 1:**
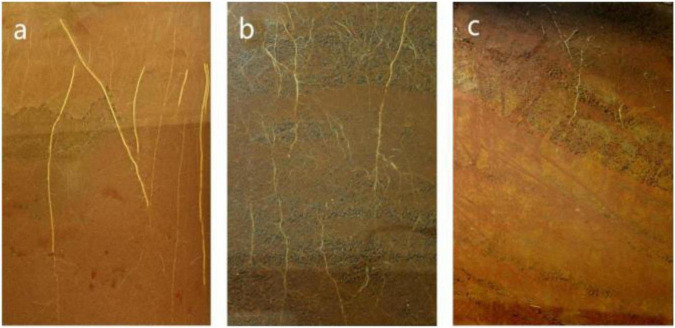
Morphological characteristics of the different soil types. **(a)** Limestone soil (LS); **(b)** paddy soil (PS); and **(c)** red soil (RS).

In this study, the flue-cured tobacco variety K326 (provided by Yuxi Zhongyan Seed Co. Ltd., Yunnan, China) was used. The experiment was conducted at the Yanhe Experimental Base, Hongta District, Yuxi City, Yunnan Province, China, from April to August 2019, at an elevation of 1,635 m. In the dry shed experiment, different soil types widely distributed in Yunnan were selected, namely, limestone soil (LS), paddy soil (PS), and red soil (RS) (Figure 1). Table 1 shows the Nutrient contents of the soils used in the experiment of three types of soil as a supplement. Each planting area had a size of 23.90 m^2^ (4.15 m × 5.76 m), and the different planting areas were divided by concrete pouring. A random block arrangement was used, with three replicates per treatment. The pest control and other field management measures were identical for each treatment and carried out in accordance with the guidelines for the production and management of high-quality tobacco leaves in Yunnan Province. After topping, three tobacco plants with uniform growth were randomly selected from each plot as the biological replicates; the roots were rinsed under running water, the root tip was preserved in liquid nitrogen (3–5 cm), and the samples were stored in a refrigerator at −80°C for the determination of various indices.

**TABLE 1:** Nutrient contents of the soils used in the experiment.

Soil type	Rhizosphere soil bulk density (g/cm^3^)	Rhizosphere soil poPODity (%)	Rhizosphere soil water content (%)	Organic matter (%)	pH	Available nitrogen (mg/kg)	Available phosphorus (mg/kg)	Available potassium (mg/kg)
LS	1.27	56.87	19.38	2.71	7.16	136.0	27.4	56.7
PS	1.42	46.26	24.90	4.37	7.8	175.8	10.1	88.2
RS	1.40	51.49	21.04	3.91	6.04	120.4	10.5	133.5

*LS, limestone soil; PS, paddy soil; RS, red soil.*

### Morphological Characteristics of the Root System

The flue-cured tobacco roots were cleaned with deionized water, scanned with a root scanner (Epson Experssion 10000XL, EU-88, Seiko Esson., Nagano-ken, japan), and the image was stored on the computer. When scanning, 10–15 mm distilled water was injected into the transparent tray to facilitate the expansion of flue-cured tobacco roots. The characteristic parameters of tobacco roots, such as root length, root surface area (RSA), root volume, root average diameter, root tip number, and root fork number, were measured using the root analysis software WinRHIZO (WinRHIZO Pro 2017, Regent Instruments Canada Inc., Quebec, Canada). Each process was repeated three times.

### Root Physiological Index

#### Root Vigor

Root vigor was determined according to Shahid et al. (2017), with slight modifications. Briefly the 0.5 of the root tip sample was incubated in a test tube with a mixture of 0.4% triphenyl tetrazolium chloride (TTC) and 0.06 mol/L phosphate buffer (pH 7.0) at 10 ml, 37°C, for 1–3 h, and the reaction was terminated after adding 2 ml of 1 mol/L sulfuric acid. Subsequently, the sample was taken out of the test tube, placed in a mortar, spiked with 3–4 ml of ethyl acetate and a small amount of quartz sand, ground, and extracted with TPF; the ground liquid was transferred to a new test tube, and the volume was brought to 10 ml with ethyl acetate. The optical density (OD) was recorded using a spectrophotometer at a wavelength of 485 nm; each process was performed in triplicate.

#### Nitrate Reductase Activity

The determination of NR activity was performed as described in Liu R. L. et al. (2016), Liu J. P. et al. (2016), with modifications. Briefly, 0.5 g of the root tip sample was placed into a test tube, and 5 ml of a mixed solution containing 100 mmol/L KH_2_PO_4_ (pH 7.5), 2% propanol, and 30 mmol/L KNO_3_. The samples were vacuumed for 5 min and incubated in the dark for 30 min in an oscillating water bath at 25°C. After incubation, 1 ml from each sample was transferred to a new test tube, and 1 ml of sulfonamide reagent, 0.02% naphthalene diamine, and 50 μl of acetonitrile were added. After incubation at room temperature for 30 min, absorbance at 540 nm was measured using a spectrophotometer. Each process was performed in triplicate.

#### Superoxide Dismutase Activity

The activity of superoxide dismutase (SOD) was determined according to Liu (2019), with modifications. For this, 0.5 g of flue-cured tobacco was ground, placed into a test tube, and mixed with 1.5 ml of 0.05 mol/L phosphate buffer (pH 7.8), 0.3 ml of 1% polyvinylpyrrolidone (PVP), and 0.3 ml of 1 mmol/L ethylenediaminetetraacetic acid (EDTA). The homogenate was centrifuged at 15,000 × *g* at 4°C for 15 min, and the supernatant was used to determine the activity of SOD. Subsequently, 250 μmol/L nitroblue tetrazolium chloride (NBT), 2.8 mmol/L N, N, N′, N′-tetramethylethylenediamine (TEMED), 22 μmol/L riboflavin, and 3 ml crude enzyme solution were added, and the mixture was incubated in the dark for 25 min. After this, the tubes were placed in a box lined with aluminum foil and irradiated at room temperature with two 20-W light-emitting diode (LED) illuminators until the SOD active band was visible. After the reaction, the absorbance values of the blank and the reaction solution were determined spectrophotometrically at 560 nm; each process was performed in triplicate.

#### Peroxidase Activity

The activity of peroxidase (POD) was determined according to Guo et al. (2018), with slight modifications. Briefly, 0.5 of the root tip sample was ground and placed into a test tube, followed by the addition of 2.9 g/ml of 0.05 mol/L phosphate buffer solution (pH 5.5). After incubation, the supernatant was centrifuged for 10 min at 5,000 *g*, and 0.1 ml of the supernatant was added to a new test tube and spiked with 1 ml of 0.05 mol/L guaiacol and 1 ml of 2% H_2_O_2_. The final volume was 5 ml, and absorbance was recorded at 470 nm; each process was carried out in triplicate.

### Protein Extraction

For this, 0.5 g of root tip tissue was ground to a powder, and 2 ml of protein lysate (7 mol/L urea, 2 mol/L thiourea, 4% SDS, 40 mmol/L Tris-HCl, pH8.5, 1 mmol/L PMSF, 2 ED mmol/LTA) was added. After mixing and incubating for 5 min on ice, a dithiothreitol (DTT) solution with a final concentration of 10 mmol/L was added. The solution was subjected to ultrasound for, centrifuged at 13,000 *g* for 20 min at 4°C, and the obtained supernatant was transferred to a new centrifuge tube. After the addition of four times the volume of the supernatant of cold acetone, the mixture was left overnight in a dark chamber at −20°C and subsequently centrifuged at 13,000 *g* for 20 min; the precipitated protein pellet was collected. The reduction reaction was carried out with 8 mol/L urea and 100 mmol/L tetraethylammonium borohydride (TEAB) (pH 8.0) in a water bath at 56°C for 30 min. Finally, iodacetamide (IAM) with a final concentration of 55 mmol/L was added, and the protein concentration was determined by the Bradford method (Bradford, 1976). Each process was performed in triplicate.

### Isobaric Tags for the Relative and Absolute Quantification Labeling

The peptides were dissolved in 0.5 mol/L TEAB and labeled with iTRAQ (AB SCIEX, United States), according to the instructions of the iTRAQ-8 standard kit. The root samples of flue-cured tobacco grown in PS, RS, and LS were labeled with iTRAQ 114, 116, and 118, respectively, at three replications. After labeling and mixing, the samples were separated by an SCX column in triplicate.

### Liquid Chromatography With Tandem Mass Spectrometry Analysis

Triple TOF 5600 (AB SCIEX, Framingham, MA, United States) + liquid chromatography-mass spectrometry (AB SCIEX, Framingham, MA, United States) was used for mass spectrometry (MS) data acquisition, and the samples were analyzed using a Triple TOF 5600 plus mass spectrometer coupled with an Eksigent nanoLC system (AB SCIEX, Framingham, MA, United States). The polypeptide sample was dissolved in 2% acetonitrile and 1% formic acid and eluted with a time gradient of 90 min and a flow rate of 300 nl/min. The two mobile phases were buffer A (2% acetonitrile, 0.1% formic acid, and 98% H_2_O) and buffer B (98% acetonitrile, 0.1% formic acid, and 2% H_2_O), and finally freeze-dried. The mass-charge ratio ranges of primary and secondary scanning were 350–1,500 and 100–1,500 *m/z*, respectively. Each process was performed in triplicate.

### Protein Identification and Quantification

For this, the data were retrieved from the Protein-PilotTM (v4.5) search engine and compared with the tobacco protein database. By further filtering the identification results of Protein-Pilot, we assumed that further quantitative identification can be carried out only at a credibility level above 95% (unused score ≥ 1.3) and a false discovery rate (FDR) below 5%. In the pairwise comparisons of flue-cured tobacco roots under three different soil types, the filter criterion of differential proteins was set as a maximum *p* of 0.05, and the difference multiple was more than 1.5 × or less than 0.83 × (fold changes). The trusted protein contains at least one unique peptide before it can be used for protein quantification.

### Bioinformatics

The differentially abundant proteins were functionally classified and annotated by Gene Ontology (GO),^1^ and their pathways were analyzed using the Kyoto Encyclopedia of Genes and Genomes (KEGG) database.^2^

### Protein Validation by Parallel Reaction Monitoring

The protein expression level and the protein function obtained by iTRAQ analysis were verified using the parallel reaction monitoring (PRM) technique. For this, the protein extract and trypsin digestion solution were prepared according to the iTRAQ technology; subsequently, the trypsin peptides were loaded on a C18 capture column for desalination, and PRM verification was carried out using a TripleTOF 5600 LC-MS/MS system (AB SCIEX, Ma limestone soil Massachusetts, United States). Collections MS1 and MS2 were analyzed in the range of 350–1,500 and 100–1,500 m/z. Some trypsin peptides were mixed and detected by MS data-dependent (DD) acquisition. Protein quality was determined by the Protein-Pilot software, and the MS data were processed using the Skyline software (spectrum library). The target peptide m/z was added to the inclusion list, the PRM collection method was established, and the PRM data collection of mixed samples was optimized and adjusted. Using the optimized PRM MS collection method, the data of each sample and PRM spectral files were collected, and the quantitative information of the proteins was obtained by analysis.

### RNA Extraction and Quantitative Reverse Transcription PCR Analysis

After extracting the total RNA from tobacco root samples with TRTzol reagent (Invitrogen, New York, NY, United States), cDNA was reverse-transcribed from 1 μg total RNA with ReverTra Ace qPCR RT kit (TOYOBO) according to the manufacturer’s instructions, using cDNA as a template for three replications. Real-time fluorescence quantitative PCR (RT-qPCR) internal reference gene actin primers were used for q-PCR amplification to verify cDNA quality, using the following conditions: denaturation at 95°C for 1 min, 95°C for 15 s, 40 cycles, followed by annealing and extension at 60°C for 30 s. The expression level of each gene was calculated and analyzed as described in Livak and Schmittgen (2013), using the 2^–ΔΔCt^ method, using three biological replicates.

### Statistical Analysis

For data analyses, SPPS18.0 (SPPS, Chicago, IL, United States) Duncan’s multiple test (Duncan’s multiple range test) and the logarithmic data processing and analysis software Origin8.0 (OriginLab. Corp., Northampton, MA, United States) were used.

## Results

### Morphological Characteristics of the Root System

**FIGURE 2:**
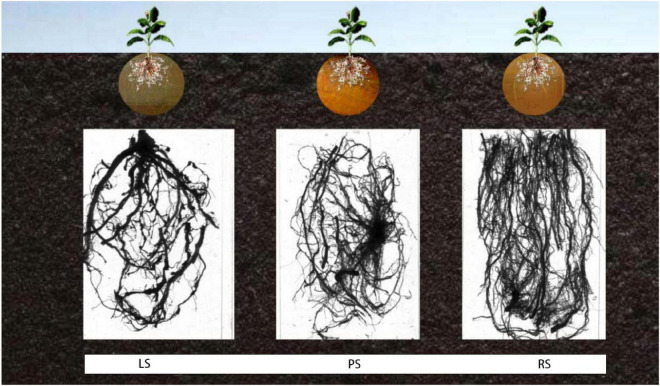
Root morphology of flue-cured tobacco under different soil treatments. LS, limestone soil; PS, paddy soil; RS, red soil.

As shown in Figure 2, irrespective of the treatment, the lateral and adventitious roots were the main parts of the root system. The root system of flue-cured tobacco in RS was well-developed, and the numbers of lateral and adventitious roots were larger than those in limestone and PS. Compared with limestone and PS, in RS, the root system was more developed, facilitating nutrient uptake.

Table 2 shows the results of the morphological analysis. Regarding root length, there were significant differences between RS and LS and between RS and PS, whereas for the RSA, there was a significant difference between RS and PS. For the root volume, there were significant differences between RS and LS and between RS and PS. The average root diameter followed the order PS > RS > LS, with significant differences between RS and LS and between RS and PS. The number of root tips differed significantly between PS and LS and between PS and RS, whereas the number of root forks differed significantly between RS and LS and between RS and PS.

**TABLE 2:** Morphological root characteristics under different soil types.

Treatment	RL/cm	RSA/cm^2^	RV/cm^3^	RAD/mm	RT	RF
LS	1,273.32 ± 107.02b	576.38 ± 59.71ab	11.07 ± 2.81b	0.81 ± 0.25b	1,918.67 ± 339.75b	16,413.33 ± 2,997.08b
PS	1,337.47 ± 126.64b	460.88 ± 60.31b	12.93 ± 4.03b	0.94 ± 0.21ab	13,456.33 ± 1254.26a	13,476.33 ± 1,254.06b
RS	1,863.25 ± 60.97a	673.05 ± 60.31a	20.13 ± 3.16a	1.21 ± 0.17a	2,984.33 ± 1254.06b	22,070.00 ± 3,869.91a

*Mean ± SE. Means within each column followed by the same letters are not significantly different at p < 0.05 based on one-way ANOVA followed by Duncan’s multiple-range test. RL, root length; RSA, root surface area; RV, root volume; RAD, root average diameter; RT, root tips; RF, root forks; LS, limestone soil; PS, paddy soil; RS, red soil.*

### Root Physiological Index

**FIGURE 3:**
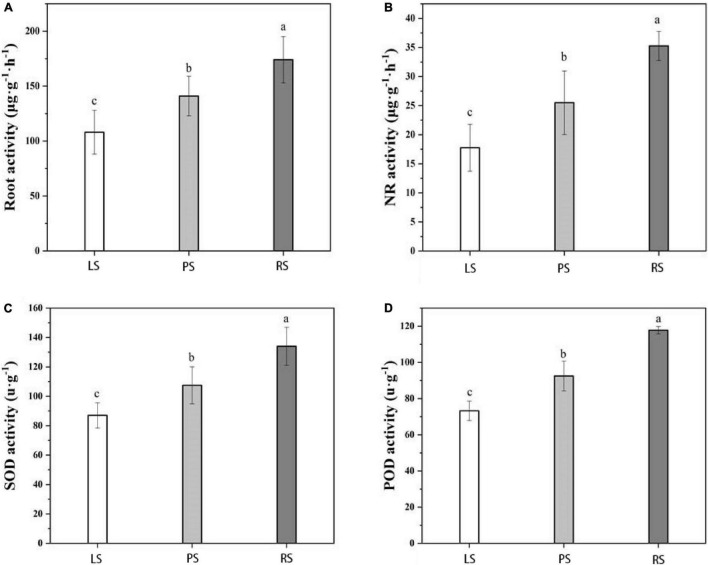
Root physiological indices of flue-cured tobacco under different soil types. **(A)** Root; **(B)** nitrate reductase (NR); **(C)** superoxide dismutase (SOD); **(D)** peroxidase (POD); Duncan’s multiple-range tests; *p* < 0.05; LS, limestone soil; PS, paddy soil; RS, red soil. Different lowercase letters showed significant difference (P < 0.05).

As shown in Figure 3, root vigor, nitrate reductase activity, SOD activity, and POD activity differed significantly under the different soil types (*p* < 0.05), following the order RS-grown plants > PS-grown plants > LS-grown plants. The root vigor of RS-grown plants was 35.23 and 21.02% higher than that of LS- and PS-grown plants, respectively. The NR activity of RS-grown plants was 51.43 and 25.71% higher than that of LS- and PS-grown plants, respectively. The SOD activity of RS-grown plants was 34.09 and 20.45% higher than that of LS- and PS-grown plants, respectively, whereas the POD activity of RS-grown plants was 37.29 and 21.19% higher than that of LS- and PS-grown plants, respectively.

### Protein Identification and Profiling

**FIGURE 4:**
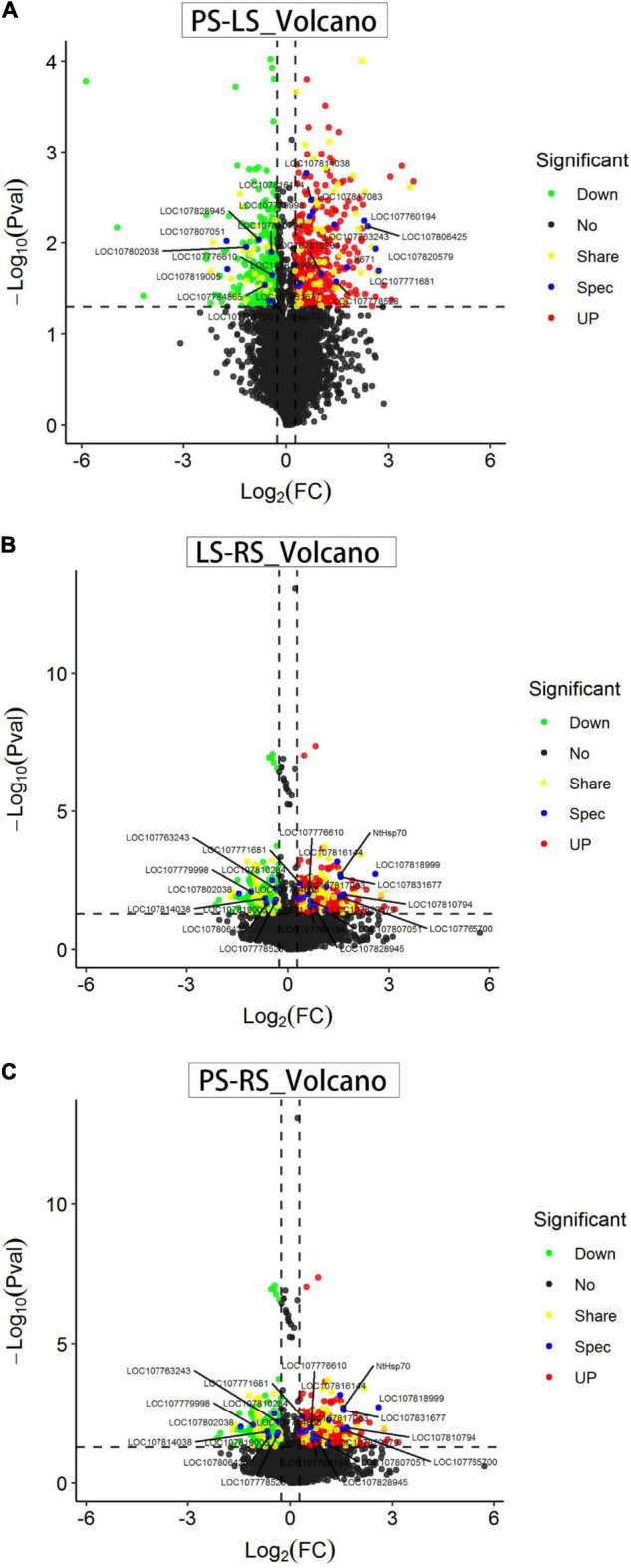
Volcano plot showing the corrected values of *p* for the changes in the patterns of all identified proteins in pairwise comparisons. **(A)** Differentially abundant proteins (DAPs) in LS vs. PS; **(B)** DAPs in LS vs. RS; and **(C)** DAPs in PS vs. RS. Red indicates significantly upregulated proteins. Green indicates significantly downregulated proteins. Black indicates proteins whose levels did not significantly change. Yellow indicates significantly share-regulated proteins. Blue indicates significantly spec-regulated proteins.

The volcanogram (Figure 4) shows the proportions of differentially abundant proteins (DAPs) of the total quantitative proteins, with log2 (FC) as the abscissa and negative logarithm-log10 (*p*) as the ordinate. In this study, 699 differentially abundant proteins were identified in PS vs. LS, such as 412 upregulated and 287 downregulated proteins (Figure 4A). When comparing LS vs. RS, we obtained 650 differentially abundant proteins, such as 291 upregulated and 359 downregulated proteins (Figure 4B). Similarly, there were 569 differentially abundant proteins between PS and RS, such as 323 upregulated and 246 downregulated proteins (Figure 4C).

**FIGURE 5:**
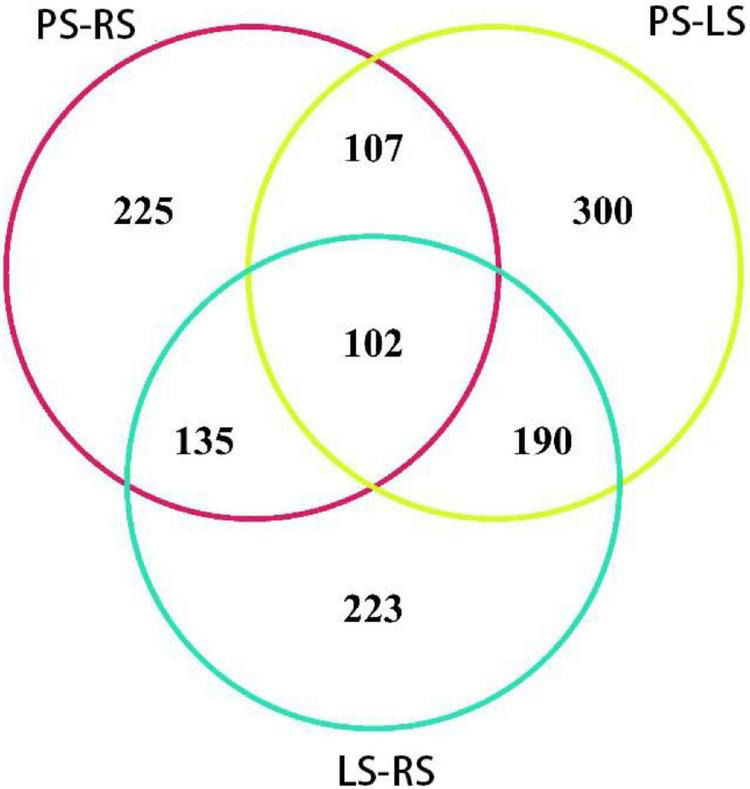
Venn diagram of DAPs from different comparison groups in tobacco root samples.

When the protein difference obtained by iTRAQ analysis was more than 1.2-fold or less than 0.83-fold, and the *p* was less than 0.05, the protein was identified as differentially expressed (significantly upregulated and downregulated protein, respectively). Among the 1,282 differential proteins in the three comparison groups, 102 proteins occurred in the three comparison groups (Figure 5). There were 432 proteins in two comparison groups, such as 190 in PS vs. LS and LS vs. RS; 135 in PS vs. LS and PS vs. RS, 107 in PS vs. LS and PS vs. RS; 748 in only one comparison group, 300 in PS vs. LS, 233 in LS vs. RS, and 225 in PS vs. RS.

### Enriched Pathways of Differentially the Abundant Proteins in Tobacco Roots

To further understand the biological functions of differential proteins in flue-cured tobacco roots in different soil types, we investigated the different pathways. In the PS vs. LS comparison group, 520 differential proteins were annotated to 103 pathways and significantly enriched in 15 metabolic pathways, based on KEGG enrichment analysis. In the LS vs. RS comparison group, 444 differential proteins were annotated to 104 pathways and significantly enriched in 18 metabolic pathways (*p* < 0.05). In the PS vs. RS comparison group, 398 differential proteins were annotated to 101 pathways and significantly enriched in 15 metabolic pathways.

### Changes in Abundances of Differential Proteins

**FIGURE 6:**
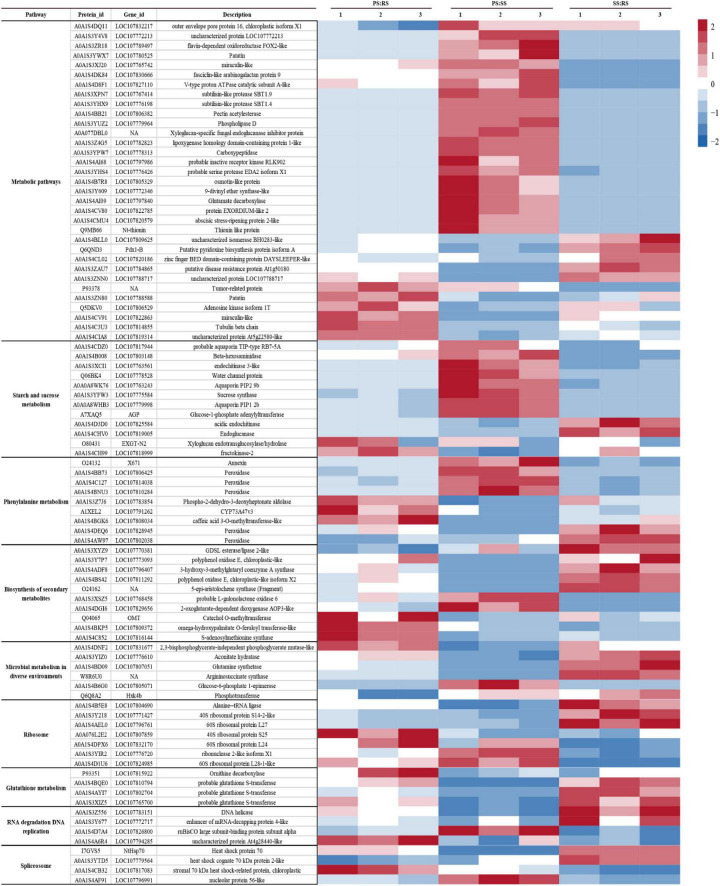
Differential protein relative expression matrix heatmap of the three groups. The color in the figure indicates the relative protein abundance level of the protein in the sample. The red color indicates that the protein has an increased protein abundance level in the sample, and the blue color represents a decreased protein abundance level.

A hierarchical cluster analysis of 102 differential proteins co-existing in the three contrasting groups was carried out, and a heatmap (Figure 6) was generated, which showed significant differences among the comparison groups. Through their abundance patterns and functional annotations, we could easily identify significant enrichment metabolic pathways in response to different soil types, indicating the rationality of the identified differential proteins and further proving the reliability of the data. In this study, nine significantly enriched metabolic pathways were detected from the 102 common differential proteins in flue-cured tobacco root samples. Numerous differential proteins were involved in starch and sucrose metabolism (12), phenylalanine metabolism (9), the biosynthesis of secondary metabolites (10), microbial metabolism in different environments (6), ribosomes (7), glutathione metabolism (4), RNA degradation (4), DNA replication (4) splice bodies (4), and others (13).

### Differentially the Abundant Proteins Involved in Defense Mechanisms

**FIGURE 7:**
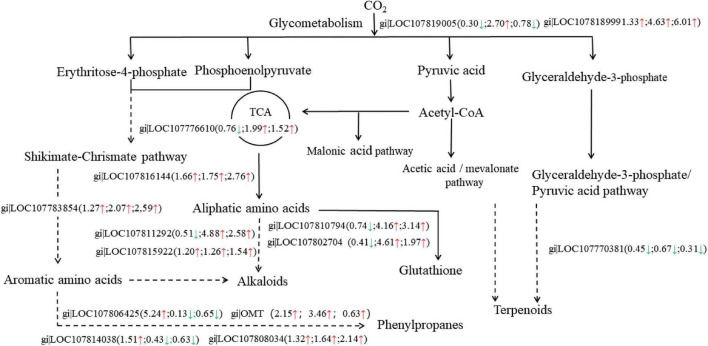
The Kyoto Encyclopedia of Genes and Genomes (KEGG) pathway of DAPs related to defense mechanisms. Upregulated proteins are marked with upward red arrows and down-regulated proteins with downward green arrows; the numbers represent the fold change. The left arrows or numbers represent the differential proteins in LS vs. PS, the middle arrows or numbers represent the differences in proteins in LS vs. RS, the right arrows or numbers represent the differences in proteins in PS vs. RS.

The iTRAQ analysis revealed that 13 proteins related to defense mechanisms were screened out from 102 common differential proteins in the three comparison groups (Figure 7). A total of eight DAPs were involved in the secondary metabolic pathway, two DAPs were involved in glucose metabolism, two in the synthesis of glutathione, and one in the synthesis of methionine. Among the 13 differential proteins related to defense mechanisms, 10 were upregulated in LS vs. RS and/or PS vs. RS, and three were down-regulated. Based on these results, we suggest that the different soil types affected the defense mechanism of flue-cured tobacco roots, and the regulatory network was more complex.

### Parallel Reaction Monitoring Verification

**FIGURE 8:**
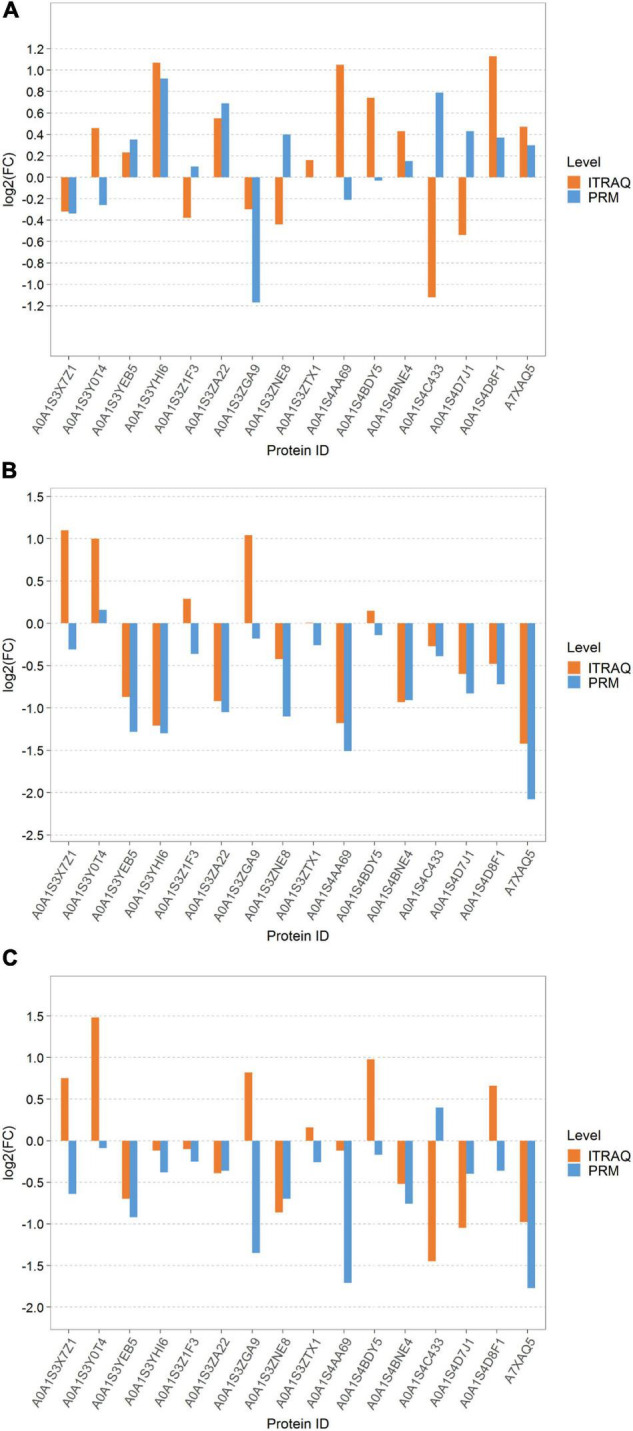
Column map of parallel reaction monitoring (PRM) quantitative protein differences among different samples. **(A)** LS vs. PS; **(B)** LS vs. RS; and **(C)** PS vs. RS. The proteins corresponding to the column on the upside of the transverse axis 0 represent upregulated proteins, the proteins corresponding to the column on the underside of the transverse axis 0 represent downregulated proteins, numbers on columns represent multiple differences (non-logarithmic), for example: if the number in the underside column is 2, the WM is two times as much as the IM in the expression level; if the number in the upside column is 2, the WM is 0.5 times as much as the IM in the expression level.

The expression levels of the top 16 target proteins were quantitatively analyzed by PRM to verify the reliability of the iTRAQ proteomic results (Figures 8A–C). We analyzed the expression levels of these 16 proteins under the three different treatments (PS vs. LS, LS vs. RS, and PS vs. RS). The results of the PRM showed that 8, 11, and 11 proteins among the three comparison groups were consistent with the results of the iTRAQ, whereas 8, 5, and 5 proteins contrasted with the results of the iTRAQ. The results showed that five proteins were consistent with the quantitative results of the iTRAQ in the three comparison groups, such as A0A1S3YEB5, A0A1S3YHI6, A0A1S3ZA22, A0A1S4BNE4, A7XAQ5, A0A1S3YEB5, A0A1S3YHI6, A0A1S3ZA22, and A0A1S4BNE4. Overall, the results were reliable and reproducible.

### Quantitative Reverse Transcription PCR Complementation

**FIGURE 9:**
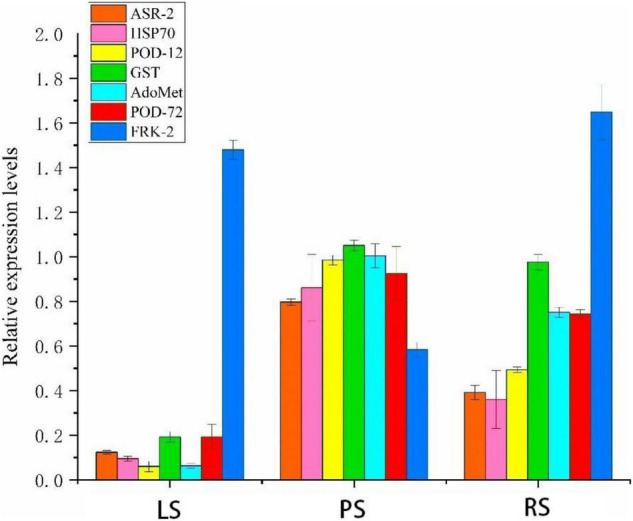
Complementation of isobaric tags for relative and absolute quantitation (iTRAQ) results by real-time quantitative PCR (qRT-PCR). Values represent the means ± SE (*n* = 3). ASR-2, aquaporin PIP1 2b; HSP70, heat shock protein 70; POD-12, peroxidase 12-like; GST, probable glutathione S-transferase; adomet, S-adenosylmethionine synthase; POD-72, peroxidase 72-like; FRK-2, fructokinase-2; LS, limestone soil; PS, paddy soil; RS, red soil.

The mRNA expression levels of seven differentially expressed key protein coding genes were detected by real-time quantitative PCR (qRT-PCR) to provide accurate data for studying the molecular mechanisms of tobacco root responses to different soil types. These seven proteins or genes are involved in the defense mechanism; the qRT-PCR data of the seven genes were consistent with the results of the iTRAQ (Figure 9).

## Discussion

### Analysis of Root Morphological Characteristics of Flue-Cured Tobacco Grown in Different Soil Types

Root morphological characteristics (root length, surface area, root volume, root average diameter, root tip number, and root fork number) play an important role in determining nutrient and water absorption efficiencies and in assessing the adaptability of a plant to stress (Zhang, 2018). The root system of flue-cured tobacco is highly diverse, with a pronounced plasticity. Based on our results, changing the rhizosphere environment of the root system can improve the morphological characteristics of the tobacco root system, thereby promoting the plant growth. Wang (2020) found that different soil types can differently affect the morphological characteristics of maize roots.

Based on previous studies, soil physical and chemical properties, temperature, nutrient content, moisture, air permeability, organic matter content, poPODity, and other factors affect the root growth of flue-cured tobacco. LS has a high sand content, light texture, fast fertilizer effect, poor ability of water and fertilizer conservation, low organic matter content, and large temperature changes; however, its air permeability and water permeability are good (Zhang, 2011). PS, as a product of farming activities, is generally subjected to long-term flooding, with a low oxygen content, a thin soil layer, and a heavy soil texture (Zang et al., 2021). Regarding red loam soil, the available potassium level is higher, the soil is stickier, the fertilizer effect is more prolonged, and the water-holding capacity is larger, and the soil pH is neutral to slightly acidic (Li et al., 2015). Li et al. (2020) indicated that acidic soil has a higher effective nutrient content, which is suitable for the growth of flue-cured tobacco.

Affected by various factors in the soil, the root morphology of flue-cured tobacco also differs with the soil type (Wang et al., 2006). It is generally believed that the formation of a longer root with a smaller diameter facilitates nutrient and water absorption. On the other hand, a thicker root system can exert greater strength to overcome the mechanical resistance of the soil and make the root system elongate, which is beneficial in more compact soil (Gong et al., 2020). In this experiment, the root length of tobacco after topping was higher under RS compared with PS and LS; the average root diameter followed the order PS > RS > LS. The root length was therefore highest under RS, with a moderate diameter and a good root system growth. However, the average root diameter under PS was larger, which may be related to the large soil capacity and compactness. In LS, root length and diameter were relatively small, although the soil texture of LS was relatively loose; the root system of flue-cured tobacco collected in this experiment was in the late growth stage, after topping. In LS, nutrient conservation was weak, and the soil did not provide sufficient nutrients for tobacco root growth. This also resulted in a small average root diameter. RSA and root volume are related to the contact area between the root system and the soil. The larger the contact area, the more water and nutrients are absorbed, enhancing plant growth (Hu, 2013).

In this study, the RSA and root volume of RS flue-cured tobacco were larger than those of LS and PS plants, suggesting that RS promoted an increase in RSA and root volume. According to a previous study, higher RSA and root volume values facilitate the drought resistance of plants (Zhang et al., 2016). Generally, the different soil types had no effects on root length, surface area, root volume, root average diameter, and root tip number of flue-cured tobacco.

### Effects of Different Soil Types on Root Physiological Indices of Flue-Cured Tobacco

Root vigor is a physiological index reflecting the metabolic level of the root system and the synthesis of various compounds. It directly affects the growth and development of plants, with a significant correlation between root vigor and the chemical composition of tobacco leaves. The higher the root vigor, the stronger the stress resistance of flue-cured tobacco plants. According to Zhao et al. (2010), in tobacco, root vigor is affected by the soil type. In our study, under RS, the root vigor was significantly higher than that under PS and LS, which could be explained by the fact that the soil water content is the main factor limiting root vigor; in LS, with the largest pore space, the water-holding capacity was lowest. In addition, high temperatures and fertilizer levels, which were encountered in PS, can inhibit root vigor. In contrast, RS had a more favorable water-holding capacity. Based on these results, RS was most suitable for the absorption and use of soil water by tobacco roots, supported the growth of aboveground parts, and created favorable conditions for high tobacco yield and quality. At the same time, topping promoted root vigor and contributed to the full yellowing of flue-cured tobacco in the later stage.

Nitrate reductase is a key enzyme in plant nitrogen metabolism. Under the action of NR, glutamine synthetase, and glutamate synthetase, nitrate nitrogen is reduced to nitrite nitrogen in roots, which is transformed into amino acids and then participates in nitrogen metabolism. The NR activity therefore reflects the level of nitrogen metabolism. Li et al. (2007), in a study on tobacco, showed that within a certain range, with increasing soil water content, the activity of NR increases, thereby promoting the plant metabolism. As different soil types differ in their water contents, water supply is also different. In this experiment, the water content was highest in RS, which therefore also promoted the NR activity of flue-cured tobacco roots.

Cell metabolism can produce reactive oxygen species (ROS), which can harm the biological system; the activity of antioxidant enzymes is therefore a key factor in active oxygen detoxification. SOD plays an important role in plant oxygen metabolism (Guo et al., 2012) and is the first line of defense against ROS oxidative damage. In addition, POD is an important antioxidant enzyme in plants (Yang et al., 2016), and in this study, the SOD and POD activities of RS flue-cured tobacco roots were lower than those under PS and LS; this is in agreement with the findings of Zhang (2018).

### Proteins Associated to Defense Mechanisms

As defense molecules, secondary metabolites can protect plants under various adverse conditions. Numerous plants can produce secondary metabolites after external stimulation, and the regulation mechanism of these metabolites involved in plant defense responses is highly complex. A variety of defense signal pathways form a defense network, which can effectively resist various biological stresses to a certain extent (Afrin et al., 2015). Lei et al. (2016) found that the change in the secondary metabolic pathway may change the composition and content of secondary metabolites. In this study, the KEGG pathway analysis showed that the biosynthesis pathway of secondary metabolites was one of the metabolic pathways of root enrichment in flue-cured tobacco under the three soil types, and hundreds of differentially abundant proteins related to secondary metabolite biosynthesis were identified. In LS vs. RS and PS vs. RS, the proportion of upregulated proteins related to secondary metabolite biosynthesis was higher than that of downregulated proteins. In the comparison of PS and LS, the opposite pattern was observed. These differential proteins may be involved in phenolic biosynthesis, terpene biosynthesis, and alkaloid synthesis, thereby improving the stress resistance of tobacco roots. The results of the KEGG annotation showed that the differential proteins were enriched in the biosynthesis of phenylalanine. Ye et al. (2001) showed that different soil physical and chemical properties had various effects on phenylalanine synthesis. In the present study, the phenylalanine biosynthesis pathway may have been involved in the response of tobacco roots to different soil types.

Phenylpropane compounds are secondary metabolites of plants and play an important role in the interaction between plant growth and the environment. Studies have shown that phenylpropanoids can prevent the invasion of pathogens by increasing the thickness of the secondary cell walls. The metabolism of phenylpropanoids provides plants with a large number of important phenolic compounds (Zhao, 2020). In our experiment, a high number of differentially expressed proteins were detected in the phenylpropanoid biosynthesis pathway. In the PS vs. LS comparison group, 37 proteins were involved in the phenylpropanoid biosynthesis pathway, of which 29 were upregulated and 8 were downregulated. We observed 42 proteins in the LS vs. RS group, of which 17 were upregulated and 25 were downregulated. In the PS vs. RS comparison groups, 24 proteins were detected, with 20 being upregulated and 14 downregulated. Based on these results, we can speculate that the phenylpropane biosynthetic pathway may be involved in the soil type response of tobacco roots. Wu et al. (2018) confirmed that phenylpropane biosynthesis metabolism is involved in the regulation of the antioxidant activity of tobacco plant cells.

In this study, 21 proteins related to defense mechanisms were screened from 102 common differential proteins in three comparison groups in which peroxidase could inactivate toxic substances and protect plants from the toxic effects of high oxygen concentrations. Glutathione S-transferase (GST) plays an important role by catalyzing the binding reaction of nucleophilic glutathione with various foreign chemicals, thereby facilitating detoxification. GST is the key enzyme of the glutathione binding reaction and catalyzes the initial step of this reaction (Song et al., 2010).

Fructokinase has also been found to be involved in the stress response of plant cells. The abundance of these proteins decreased in the comparison group PS vs. LS and increased in the LS vs. RS and PS vs. RS groups. The pathways of 13 differential proteins involved in stress defense were analyzed; among them, 8 differential proteins were involved in secondary metabolism, 2 in glucose metabolism, 2 in glutathione synthesis, and 1 in methionine synthesis. Among the 14 differential proteins related to defense mechanisms, 10 were upregulated in LS vs. RS and/or PS vs. RS, and 3 proteins were downregulated. Based on these results, the defense mechanism of flue-cured tobacco roots in different soil types is highly complex, with an even more complex regulatory network. The above results lead us to infer that compared with LS and PS, the resistance of tobacco roots to stress was higher in RS, in addition to the well-developed root system in RS, providing enhanced resistance.

### Proteins Linked to Carbohydrates and Energy Metabolism

Carbohydrate and energy metabolism is one of the most important biological metabolisms of flue-cured tobacco roots in response to different soil types. The pathways related to carbohydrate and energy metabolism include the glycolysis gluconeogenesis pathway, the starch and sucrose metabolism pathway, the TCA cycle, and the pentose phosphate metabolism pathway, among others. In plants, sucrose synthase is a key enzyme in sucrose metabolism and can catalyze the decomposition and synthesis of sucrose; it also plays an important role in starch synthesis, stress resistance, and plant growth (Li and Kong, 2015). In our study, we found three differentially expressed sucrose synthase proteins (A0A1S3YFR7, A0A1S4ACT4, and A0A1S3ZPV3) in PS vs. LS, LS vs. RS, and PS vs. RS; they were significantly downregulated in the PS vs. LS comparison group and upregulated in LS vs. RS and PS vs. RS. This indicates that the sucrose metabolism of tobacco roots may be affected by different soil types and that sucrose synthase may play a key role in the regulation of carbon metabolism and nitrogen fixation. The upregulated expression of sucrose synthase under RS suggests that the activity of the sucrose metabolism pathway in flue-cured tobacco roots was higher in RS.

Based on a previous study, the TCA cycle is the main way for plants to obtain energy, providing the carbon skeleton for plants to synthesize various substances (Wang et al., 2019). Several proteins involved in the TCA cycle, such as citrate synthase (W8SRJ8), were upregulated in PS vs. RS. Two malate dehydrogenases (A0A1S3YXG6 and A0A1S3ZI08) in PS vs. LS were upregulated, whereas succinate dehydrogenase (A0A1S4AC19) was downregulated. Fumarate hydratase (A0A1S3WYC1) and succinate-CoA ligase (A0A1S3XSQ4) in LS and RS were upregulated. Compared with LS and PS, the expression of proteins related to the TCA cycle was upregulated under the condition of RS, which resulted in the provision or larger amounts of energy.

In this study, we selected 10 proteins related to carbohydrate and energy metabolism, out of 102 common differential proteins. For example, glucose-6-phosphate and glucose-1-phosphate adenylate transferase are involved in carbohydrate transport and metabolism, aconitine hydratase is involved in the TCA cycle, and other proteins are involved in carbohydrate transport and metabolism. Therefore, the biological processes and pathways related to sugar and energy metabolism may be important factors regulating the adaptation of flue-cured tobacco roots to different soil types.

### Proteins Linked to Protein Synthesis Ability

Since proteins are the direct executors of various biological processes, protein synthesis is directly related to the general activities of plants (Hu et al., 2018). Ribosomes play an important role in protein translation and synthesis. In this study, 8 ribosome-related proteins were screened from the 102 common differential proteins in the three comparison groups, of which 5 were upregulated in LS vs. RS and PS vs. RS and 3 in PS vs. LS. They were 40S ribosomal proteins (40S ribosomal protein [A0A076L2E2 and A0A1S3Y218]), 60S ribosomal protein (60S ribosomal protein [A0A1S4DPX6, A0A1S4AEL0, and A0A1S4D1U6]), alanine-tRNA ligase (Alanine–tRNA ligase [A0A1S4B5E8]), and ribonuclease (ribonuclease [A0A1S3YIR2]). Among all differential proteins determined, 17 and 20 were upregulated in the ribosomal pathway in LS vs. PS and LS vs. RS, respectively. These upregulated proteins may promote protein synthesis, indicating that the protein synthesis of flue-cured tobacco roots under PS and RS conditions is higher than that under LS. Similar results have been obtained in a study investigating tobacco roots under two cropping systems (You et al., 2014).

## Conclusion

Based on determining the morphological characteristics and physiological indices of flue-cured tobacco roots, an iTRAQ technique was used to study the differential changes in root proteins under different soil types (LS, PS, and RS). The results showed that root length, RSA, and root volume were higher in RS than in LS and PS, indicating that RS was most suitable for tobacco growth. The soil type significantly changed the proteomic profiles of tobacco roots. The stress resistance, carbohydrate and energy metabolism, and protein synthesis of RS flue-cured tobacco roots were higher than those in LS and PS. Based on PRM and qRT-PCR analysis, the results of iTRAQ proteomics were correct and reliable. The response mechanism of flue-cured tobacco roots to different soil types could be revealed at the protein level.

## Data Availability Statement

The data presented in the study are deposited in the Integrated Proteome Resources repository, accession number IPX0004001000.

## Ethics Statement

Written informed consent for participation in this study was obtained from the participants.

## Author Contributions

RY and YC: conceptualization. RY: data curation, methodology, and writing–original draft. YC: funding acquisition, project administration, supervision, and validation. JlL: writing–review and editing. All authors contributed to the article and approved the submitted version.

## Conflict of Interest

RY was employed by Shiyan Branch of Hubei Tobacco Company. SS and YL was employed by Research Center of Hubei Tobacco Industrial Co., Ltd. XL was employed by Hunan Zhangjiajie Municipal Tobacco Co.

The remaining authors declare that the research was conducted in the absence of any commercial or financial relationships that could be construed as a potential conflict of interest.

## Publisher’s Note

All claims expressed in this article are solely those of the authors and do not necessarily represent those of their affiliated organizations, or those of the publisher, the editors and the reviewers. Any product that may be evaluated in this article, or claim that may be made by its manufacturer, is not guaranteed or endorsed by the publisher.

## Footnotes

1
http://www.geneontology.org

2
http://www.genome.jp/kegg/

## References

[B68] Afrin S. Huang J. J. Luo Z. Y. (2015). JA-mediated transcriptional regulation of secondary metabolism in medicinal plants. *Sci. Bull.* 60:1062. 10.1007/s11434-015-0813-0

[B69] Anderson N. L. Matheson A. D. Steiner S. (2000). Proteomics: applications in basic and applied biology. *Curr. Opin. Biotechnol.* 11 408–412. 10.1016/S0958-1669(00)00118-X 10975462

[B1] Bradford M. M. (1976). A rapid and sensitive method for the quantitation of microgram quantities of protein utilizing the principle of protein-dye binding. *Anal. Biochem.* 72 248–254. 10.1016/0003-2697(76)90527-3 942051

[B2] Cai X. Z. (2014). *Comparative Proteomics Research in the Application of Differentially Expressed Protein Detection.* Taichung: China Medical University.

[B3] Cai Y. Z. Zhou P. X. Zhang L. Wang Z. Xu Q. H. Yang H. W. (2015). Effects of climate conditions on photosynthetic rate and protein expression in Yunyan 87 leaves atvigorous growth stage. *Chin. J. Tobac.* 87:10. 10.16472/j.chinatobacco.2013.420

[B4] Chen L. H. Cheng Z. Z. Xu M. Zheng J. G. (2017). Research advance in iTRAQ technology and its application in proteomics of rice. *China Agricult. Sci. Technol. Bull.* 19:10. 10.13304/j.nykjdb.2017.0224

[B5] Chen Q. Liu Y. G. Yang X. Li Y. (2005). Progress in proteomics research. *J. Southw. Univ. National. Nat. Sci. Edit.* 031 257–260.

[B6] Chen R. X. Yang H. Q. Zhao S. Y. Li H. G. Cheng J. P. Fang M. (2012). Effects of soil types on growth and quality characteristics of flue-cured tobacco. *Chin. Tobac. Sci.* 33:6.

[B7] Chen Y. J. Zhou J. H. He W. Yang H. Q. Yang Z. Y. Wang L. (2010). Influences of different hilling time on the development and activity of adventitious roots of tobacco. *Hunan Agricult. Sci.* 9:5. 10.3969/j.issn.1006-060X.2010.17.010

[B8] Chen Z. Y. Bi T. Wu X. X. (2012). Effects of reduced UV-B radiation on the variation of flue-cured tobacco proteome. *Chin. J. Appl. Ecol.* 31:1129–1135.

[B9] Gao J. H. Zhou Q. M. Jin Y. (2007). Research advances in root system of flue-cured tobacco. *China Agricult. Bull.* 23:3. 10.3969/j.issn.1000-6850.2007.07.038

[B10] Gong Z. Xiong L. Shi H. Yang S. Zhu J. K. (2020). Plant abiotic stress response and nutrient use efficiency. Science China. *Life Sci.* 63:5. 10.1007/s11427-020-1683-x 32246404

[B11] Guo P. G. Chen J. J. Li R. H. (2000). Effects of pH values on the activity of roots and chemical compositions of the cured leaves in flue-cured tobacco. *Chin. Agricult. Sci.* 33 39–45.

[B12] Guo Y. Y. Yu H. Y. Yang M. M. Kong D. S. Zhang Y. J. (2018). Effect of drought stress on lipid reroxidation, osmotic adjustment and antioxidant enzyme activity of leaves and roots of Lycium ruthenicum Murr. Seedling. *Russ. J. Plant Physiol.* 65 244–250. 10.1134/S1021443718020127

[B13] Guo Z. S. Cui B. W. Lu Y. G. (2012). Effect of water stress on growth and chemical quality during the different growth period of flue-cured tobacco. *Guangd. Agricult. Sci.* 06 41–44. 10.3969/j.issn.1004-874X.2012.06.014

[B14] Han J. F. Zhang X. T. (1992). Effects of soil moisture on root development and Vigor of roots of flue-cured tobacco. *Chin. Tobac.* 000 14–17. 10.13496/j.issn.1007-5119.1992.03.004

[B15] He F. L. Wang M. Q. (1980). The growth physiology of rice roots. *Plant Physiol. Newsletter* 03 23–28. 10.13592/j.cnki.ppj.1980.03.005

[B16] Hou D. P. Yu C. Liu H. L. Cai H. Zhang Y. X. Zhu Q. Q. (2018). Characteristics of high yield and high efficiency root and regulation in rice. *Chinese Rice* 24:6.

[B17] Hou J. M. (2003). Study on the relationship between root development and yield and quality of flue-cured tobacco. *China Tobac. Sci.* 24:3. 10.3969/j.issn.1007-5119.2003.02.005

[B18] Hu R. Zhu X. Xiang S. Zhang X. Liu Z. Zhu L. (2018). Comparative proteomic analysis reveals differential protein and energy metabolisms from two tobacco cultivars in response to cold stress. *Acta Physiolog. Plant.* 40:19. 10.1007/s11738-017-2582-7

[B19] Hu W. (2013). *Effects of soil bulk density on tobacco growth and soil chemistry properties of tobacco growing.* Yunnan: Kunming University of Science and Technology.

[B20] Hu W. W. (2015). *The response mechanisms of root growth to phosphorus stress and auxin treatment in tobacco.* Hunan: Hunan Agricultural University.

[B21] Huang J. (2008). *Reaserch on main influencing factors of adventitious roots development and related physiological functions of flue-cured tobacoo.* Hunan: Hunan Agricultural University.

[B22] Lei B. Qin J. Liao C. S. Zhao H. N. Ding F. Z. Ren Z. (2016). Comparative iTRAQ analysis of the leaf proteome of tobacco (*Nicotina tabacum L.*) plants grown in two different ecological regions. *Ecol. Env. Monit. Three Gorges* 1:7. 10.19478/j.cnki.2096-2347.2016.03.04

[B23] Li J. P. Chen Z. G. Yang Y. H. Cai H. Y. (2007). Study on the adequate soil moisture indexes of flue-cured tobacco irrigation based on some physiological indexes. *J. Irrig. Drain.* 26:4. 10.3969/j.issn.1672-3317.2007.01.025

[B24] Li L. N. Kong J. Q. (2015). Gene organization, function and application of plant sucrose synthase. *Chin. J. Biochem. Molecul. Biol.* 31:904–913. 10.13865/j.cnki.cjbmb.2015.09.03

[B25] Li X. Pan X. Z. Li B. J. Zhang D. M. (2020). Characteristics and correlation of paddy soil nutrients in Chengmai County. *Trop. Agricult. Sci.* 40:7.

[B26] Li Z. P. Liu M. Jiang C. Y. (2015). Decomposition, accumulation and distribution of soil organic matter in typical red soil region of China. *Soil* 47:220–228. 10.13758/j.cnki.tr.2015.02.005

[B27] Lin S. F. Zhang T. Shi Y. W. Wang D. M. Wang Z. H. Yang Z. X. (2012). Comparison of protein extraction methods for two-dimensional electrophoresis in tobacco root. *Guizhou Agricult. Sci.* 40:4. 10.3969/j.issn.1001-3601.2012.08.022

[B28] Liu J. P. Fan X. You M. H. Wang S. S. Zong R. X. (2016). Changes in sugar, pyruvic acid content and nitrate reductase activity of Elymus sibiricus reproductive branches during seed development. *J. Grass Indust.* 25:9. 10.11686/cyxb2015502

[B29] Liu R. L. Wang Y. Y. Qin G. Z. Tian S. P. (2016). iTRAQ-based quantitative proteomic analysis reveals the role of the tonoplast in fruit senescence. *J. Prot.* 146 80–89. 10.1016/j.jprot.2016.06.031 27371350

[B30] Liu W. Q. Jiang Z. X. Guo H. X. Liu J. L. (2004). Effect of different nitrogen forms on the microbial number in rhizosphere of flue-cured tobacco planted in yellow cinnamon soil and fluvo-aquic soil. *Soil Bull.* 35:43–47. 10.19336/j.cnki.trtb.2004.01.011

[B31] Liu Y. W. (2019). *Extraction and Purification of Superoxide Dismutase from Phyllanthus emblica L.* and Effects of Flavonoids and Light on Activity. Changchun: Jilin Agricultural University. 10.27163/d.cnki.gjlnu.2019.000341

[B32] Livak K. J. Schmittgen T. D. (2013). Analysis of relative gene expression data using real-time quantitative PCR and the 2(-Delta Delta C(T)) Method. *Methods* 25 402–408. 10.1006/meth.2001.1262 11846609

[B33] Ma X. M. Wang X. C. Ni J. H. Liu G. S. (2003). Characteristics of tobacco root development on different types of soil. *Chin. J. Tobac.* 9 39–44. 10.3321/j.issn:1004-5708.2003.01.009 30704229

[B34] Meng L. B. Huo H. T. (2005). The research of protein custer and its academic tendency. *J. Harb. Univ.* 26:7. 10.3969/j.issn.1004-5856.2005.10.033

[B35] Nagel K. A. Kastenholz B. Jahnke S. Dusschoten D. V. Aach T. Mühlich M. (2009). Temperature responses of roots: impact on growth, root system architecture and implications for phenotyping. *Funct. Plant Biol.* 36:9184. 10.1071/FP09184 32688706

[B36] Ruan S. L. Ma H. S. Wang S. H. Xin Y. Qian L. H. Tong J. X. (2006). Advances in plant proteomics-I. key techniques of proteome. *Hereditas* 2006 1472–1486. 10.16288/j.yczz.2006.11.025 17098721

[B37] Sascha W. Elizabeth S. Jürgen K. V. (2020). Same same, but different: growth responses of primary and lateral roots. *J. Exp. Bot.* 8:8. 10.1093/jxb/eraa027 31956903 PMC7178446

[B70] Shahid M. Shukla A. K. Nayak A. K. Tripathi R. Gautam P. (2017). Vigor of roots and antioxidant enzyme activities of rice cultivars under different iron toxicity mitigation options. *J. Ind. Soc. Soil Sci.* 65:341. 10.5958/0974-0228.2017.00040.8

[B38] Shen A. L. (1994). A comparative study on the fertilizer supply performance of yellow cinnamon soil and fluvo-aquic soil in Henan Province. *Soil Bull.* 28–30. 10.19336/j.cnki.trtb.1994.01.010

[B39] Song C. Q. Miu H. F. Zhu B. Tong S. M. Li Z. Y. Hou H. S. (2010). The role of plant glutathione-S-transferase in phytoremediation. *Anhui Agricult. Bull.* 16 56–110. 10.3969/j.issn.1007-7731.2010.07.024

[B40] Sun Y. W. Jiang Y. He F. C. (2005). Advance in differential proteomics research. *Life Sci.* 17:4. 10.3969/j.issn.1004-0374.2005.02.007

[B41] Swinbanks (1995). Government backs proteome proposal. *Nature* 378 653–653. 10.1038/378653b0 7501000

[B42] Wang D. Y. Luo G. M. Liu P. P. Zhou L. Xu Y. Y. Chen Y. T. (2019). Advances in transcriptome research on plant response to waterlogging. *Biotechnol. Bull.* 35:6. 10.13560/j.cnki.biotech.bull.1985.2019-0312

[B43] Wang H. Ding W. Xu Z. C. Shi J. X. Zhang C. Y. (2006). Difference of nutrients between purple soil and other soil types in guizhou tobacco-growing areas. *Guizhou Agricult. Sci.* 034 22–25. 10.3969/j.issn.1001-3601.2006.03.008

[B44] Wang N. (2020). *Study on the effects of different fertilization on the root morphological characteristics of maize in three typical farmland.* Mudanjiang: Mudanjiang normal University. 10.27757/d.cnki.gmdjs.2020.000138

[B45] Wang W. W. Xi F. H. Yang S. F. Jiang L. F. Wang F. J. (2016). Progress on nicotine metabolism regulation in tobacco. *Subtrop. Agricult. Res.* 12:6. 10.13321/j.cnki.subtr0p.agric.res.2016.01.010

[B46] Wilkins M. R. Sanchez J. C. Gooley A. A. Appel R. D. Humphery-Smith I. Hochstrasser D. F. (1996). Progress with proteome projects: why all proteins expressed by a genome should be identified and how to do it. *Biotechnol. Genet. Eng. Rev.* 13 19–50. 10.1080/02648725.1996.10647923 8948108

[B47] Wu H. Y. Xi Q. L. Li X. H. Xue G. Yang T. Z. (2018). Study on root characteristics and their influences on rhizosphere potassium content of different flue-cured tobacco genotypes. *China Agricult. Sci. Technol. Bull.* 20:9. 10.13304/j.nykjdb.2017.0675

[B48] Wu Q. Y. Lin Y. L. Sun Y. H. Wei Q. H. Liu P. T. Li X. F. (2021). Research progress on effects of root exudates on plant growth and soil nutrient uptake. *Chin. J. Grassl.* 43 97–104.

[B49] Xiang F. Ye H. Chen R. B. Fu Q. Li L. J. (2010). N, N-dimethyl leucines as novel isobaric tandem mass tags for quantitative proteomics and peptidomics. *Analy. Chem.* 82:2817. 10.1021/ac902778d 20218596 PMC2859709

[B50] Xie Y. Y. Cheng C. H. Xia C. Jin H. Q. Wu J. Z. Liu B. L. (2010). Effects of soil types on quality of flue-cured tobacco in liangshan prefecture. *Anhui Agricult. Sci.* 2010:5. 10.3969/j.issn.0517-6611.2010.36.069

[B51] Xu M. L. Fan X. H. (1997). Root responses and rhizosphere effects of different plants under nutritional stress. *Soil* 29:5.

[B52] Xu X. Y. Sun W. S. Li Z. H. Li J. C. (2004). Synthesis of nicotine of tobacco roots and effects of pH values on the roots growth and quality of tobacco. *J. Anhui Agricult. Univ.* 31:5. 10.3969/j.issn.1672-352X.2004.03.013

[B53] Yang P. Cheng Z. M. Chen X. S. Xiang J. Y. Cai Y. Huang S. (2017). Effects of soil moisture regulation on flue-cured tobacco root system, plant morphology and physiological characteristics. *Guizhou Agricult. Sci.* 45:3. 10.3969/j.issn.1001-3601.2017.09.006

[B54] Yang S. Y. Chen X. Y. Hui W. K. Ren Y. Ma L. (2016). Progress in responses of antioxidant enzyme systems in plant to environmental stresses. *J. Fuj. Agricult. Forest. Univ.* 45 481–489. 10.13323/j.cnki.j.fafu(nat.sci.).2016.05.001

[B55] Yao S. X. (2015). *Effective stable isotope labeling with ammonium nitrate-15N in rice seedlings for quantitavie proteomic analysis.* Beijing: Qinghua Univercity. 10.1093/mp/ssu089 25122698

[B56] Ye Y. Lu C. Y. Tan F. Y. (2001). Effects of soil texture and light on growth and physiology parameters in *Kandelia candel*. *J. Plant Ecol.* 25:42–49.

[B57] You L. H. Chen D. M. Huang J. W. Tang L. N. Xu Z. B. Zhang C. Y. (2014). Analysis of root differential expression proteins of nicotiana tabacum under two planting systems. *Chin. Tobac. Sci.* 35:7. 10.13496/j.issn.1007-5119.2014.01.017

[B58] Zang X. Zhou Z. Zhang T. Wang X. Ding C. (2021). Aging of exogenous arsenic in flooded paddy soils: Characteristics and predictive models. *Environ. Poll.* 274:116561. 10.1016/j.envpol.2021.116561 33529895

[B59] Zeng D. W. Zhu K. L. Pei X. D. Wang J. W. Yang K. (2015). Review of fertilization effects on tobacco root growth. *South Agricult. Sci.* 2015:43. 10.16498/j.cnki.hnnykx.2015.06.043

[B60] Zhang L. Yin H. R. Zhang Y. Lu H. J. (2014). Advancements in bio-mass spectrometry based quantitative proteomics. *Anal. Test.* T*echniq. Instrum.* 20 139–147.

[B61] Zhang Q. (2018). Effects of rare earth fertilizer on root development during flue cured tobacco growth. *Shanxi Agricult. Sci.* 46:6.

[B62] Zhang X. D. Wang Z. W. Han Q. F. Wang Z. Y. Min A. C. Jia Z. K. (2016). Effects of water stress on the root structure and physiological characteristics of early-stage maize. *Acta Ecol. Sin.* 36 2969–2977. 10.5846/stxb201409181852

[B63] Zhang X. S. Lu J. (2021). Research status of the interaction between plant root distribution and rhizosphere microecology. *Jilin Forest. Sci. Technol.* 50 36–42. 10.16115/j.cnki.issn.1005-7129.2021.06.011

[B64] Zhang Z. K. (2011). *Study the spatial of soil properties indifferent level stream of three gorges reservoir typical basin.* Chongqing: Southwest University, 10.7666/d.y1882813

[B65] Zhao H. Zhao M. Q. Cheng Y. Y. Wang W. J. Lu Y. (2010). Change of different soil types on microorganisms and enzyme activity of the rhizosphere and non-rhizosphere of nanyang tobacco growing area in henan province. *Soil Bull.* 41:1057–1063. 10.19336/j.cnki.trtb.2010.05.007

[B66] Zhao Y. (2020). *Transcriptome analysis reveals the mechanism of nitric oxideon regulating drought stress response in medicago satival.* Gansu: Gansu Agricultural University. 10.27025/d.cnki.ggsnu.2020.000021

[B67] Zhou J. H. Fang X. D. Duan C. Z. Cai X. J. Yu J. B. (1999). Study on adaptive capacity to rhizosphere pH in different flue-cured tobacco varieties. *Chin. Agricult. Sci.* 32:105107. 10.3321/j.issn:0578-1752.1999.03.018 30704229

